# Supplementary material for: Effectiveness and Safety of Ayurvedic Medicines in Type 2 Diabetes Mellitus Management: A Systematic Review and Meta-Analysis
Source: Front Pharmacol. 2022 Jun 8;13:821810. doi: 10.3389/fphar.2022.821810 (PMC9213670; doi:10.3389/fphar.2022.821810)
Supplement: Supplementary file 1 [file DataSheet1.zip › Supplementary Material/Forest and Funnel Plots/Zingiber officinale/Insulin resistance.pdf]

Zingiber officinale - Insulin resistance

Study	Intervention N	Intervention Mean	Intervention SD	Comparator N	Comparator Mean	Comparator SD	Mean Diff. with 95% CI	Weight (%)
Mahluji 2013	28	3.90	1.00	30	4.50	1.80	-0.60 [-1.36, 0.16]	17.41
Arablou 2014a, Arablou 2014b	33	1.30	0.50	30	2.40	1.40	-1.10 [-1.61, -0.59]	22.92
Mozaffari-Khosravi 2014, Talaei 2017, Talaei 2018	40	0.95	0.74	41	1.00	0.74	-0.05 [-0.37, 0.27]	27.21
Shidfar 2015	22	1.58	1.00	23	2.53	1.80	-0.95 [-1.81, -0.09]	15.51
Carvalho 2020	47	3.21	1.90	56	3.20	2.10	0.01 [-0.77, 0.79]	16.95
**Overall**							-0.52 [-0.99, -0.04]	

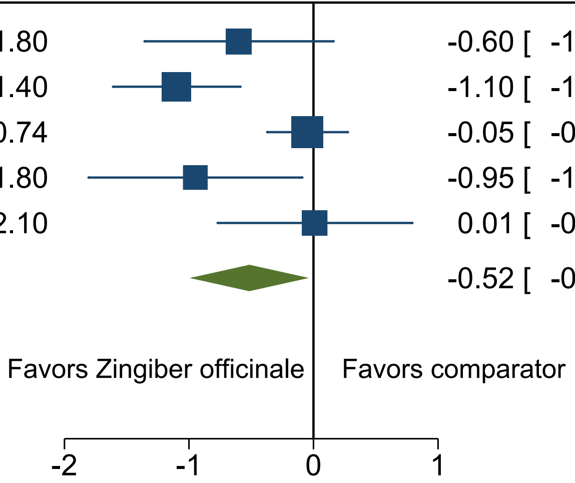

Heterogeneity: $\tau^2 = 0.19$, $I^2 = 68.50\%$, $H^2 = 3.17$

Test of $\theta_i = \theta_j$: Q(4) = 14.68, p = 0.01

Test of $\theta = 0$: z = -2.12, p = 0.03

Random-effects REML model
